# Atypical Presentation of Drug Reaction With Eosinophilia and Systemic Symptoms (DRESS) in a Patient on Pembrolizumab: A Case Report

**DOI:** 10.7759/cureus.59804

**Published:** 2024-05-07

**Authors:** Hillary Tran, Geetika Bhatt

**Affiliations:** 1 Anesthesiology, Lake Erie College of Osteopathic Medicine, Jacksonville, USA; 2 Hematology and Medical Oncology, Baptist Health-Jacksonville, Jacksonville, USA

**Keywords:** drug reaction with eosinophilia and systemic symptoms (dress), pembrolizumab side effect, pembrolizumab cutaneous side effect, immunotherapy side effects, cancer immunotherapy

## Abstract

Pembrolizumab is an immune checkpoint inhibitor that has been associated with numerous immune-mediated adverse effects. Several of these cutaneous side effects may include bullous pemphigoid, Stevens-Johnson syndrome, and drug reaction with eosinophilia and systemic symptoms (DRESS). Other case reports have reported DRESS as a rare side effect of immune checkpoint inhibitors but due to its variable presentation and similarities with other cutaneous diseases, it has proven to be a diagnostic challenge. In addition, no effective methods have been developed to monitor for such adverse skin reactions in patients on immunotherapy. Here, we report a diagnostic challenging case of pembrolizumab-induced blistering lesions that were initially treated as suspected Herpes zoster and/or bullous pemphigoid but further pathology was consistent with DRESS.

## Introduction

Immune checkpoint inhibitors (ICIs), such as pembrolizumab, have been approved for the treatment of various malignancies, which have led to improved survival rates in many oncological cases [1]. Checkpoint proteins, such as programmed death-1 (PD-1) and cytotoxic T lymphocyte antigen-4 (CTLA-4), can bind with their ligands to prevent the activation of T cells, leading to increased proliferation of tumor cells [1]. ICIs block this interaction of checkpoint proteins with their ligands, allowing for the activation of T lymphocytes to help reduce the tumor burden [1,2]. However, dermatological side effects are commonly associated with the use of ICIs. Dermatological adverse reactions associated with ICIs can range from mild rashes and blisters to more severe cutaneous manifestations, such as bullous pemphigoid, Stevens-Johnson syndrome (SJS), or toxic epidermal necrolysis (TEN), drug reaction with eosinophilia and systemic symptoms (DRESS), and other conditions [3]. In particular, DRESS syndrome is a delayed hypersensitivity reaction and immune-related adverse event that can be missed due to its nonspecific manifestations [4]. DRESS typically presents with eosinophilia, fever, diffuse rashes, pruritis, and papular or pustular erythema but its clinical presentation is variable. We report a case of squamous cell carcinoma treated with immunotherapy with pembrolizumab, who presented with blistering lesions that were thought to be secondary to Herpes zoster and/or bullous pemphigoid, but pathology revealed that these side effects were due to DRESS.

## Case presentation

A 69-year-old male was initially diagnosed with squamous cell carcinoma (SCC) of the tongue in September 2003 after presenting with a lesion on the left base of his tongue. He was treated with left partial glossectomy, radical neck dissection, and subsequent radiotherapy. He then presented again in August 2021 with progressive dysphagia and voice changes. Further workup revealed a left supraglottic mass, and he was diagnosed with recurrent SCC of the supraglottis after a biopsy. The patient declined any further surgery and opted for systemic palliative treatment. He was managed with palliative chemotherapy with carboplatin/paclitaxel/pembrolizumab for four cycles, followed by maintenance cycles of pembrolizumab monotherapy.

The patient was doing well on maintenance cycle 26 of pembrolizumab until he presented to the emergency department (ED) in July 2023 with a vesicular rash on his right chest. He was initially diagnosed with Herpes zoster and provided a seven-day course of valacyclovir with improvement of the rash. However, he developed right upper extremity swelling and new lesions on his hands, arms, and across his trunk approximately two to three days after completing a course of valacyclovir. He then presented again to the ED and was prescribed another course of valacyclovir along with antibiotics due to the suspicion of cellulitis secondary to a suspected disseminated zoster.

**Figure 1:**
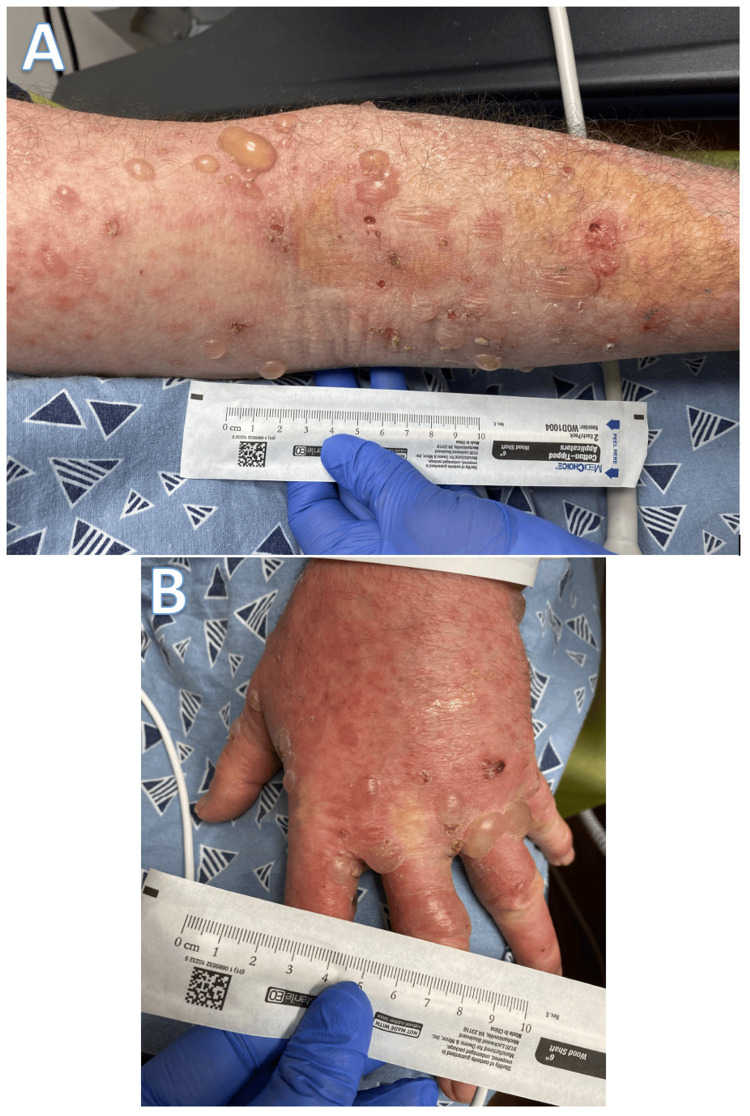
Erythematous and blistering rashes on the upper extremities (A) Demonstrates progressive blisters on the left arm, (B) Shows another view of the blisters on the left hand

He presented again to the ED three days after his previous visit due to progressive, widespread, blistering lesions across his entire body, which are shown in the images below (Figure 1). The patient also noted nausea, weakness, watery eyes, and diarrhea at the time. Labs at the time of presentation revealed notably elevated WBCs of 17.23 K/uL (Table 1). Discussion for transfer to the closest burn center was discussed with the patient, however, this was deferred due to lack of availability, and he was then admitted for further care. Differential diagnoses at the time included pembrolizumab-induced bullous pemphigoid, disseminated zoster, or both concurrently. He was subsequently started on oral prednisone and IV hydration along with a course of IV acyclovir, as both diagnoses could not be ruled out prior to the biopsy. Doxycycline was added to his regimen for cellulitis secondary to suspected bullous pemphigoid.

**Table 1:** Initial complete blood count (CBC) notable for a 17.23 K/uL white blood cell count WBC: white blood cell; RBC: red blood cell; HGB: hemoglobin; HCT: hematocrit; MCV: mean corpuscular volume; MCH: mean corpuscular hemoglobin; MCHC: mean corpuscular hemoglobin concentration; RDW: red blood cell distribution width; PLT: platelet; MPV: mean platelet volume

Component	Patient’s Value	Reference Range
WBC	17.23 (H)	3.8 - 10.8 thousand/uL
RBC	4.97	3.80 - 5.10 million/uL
HGB	15.2	11.7 - 15.5 g/dL
HCT	45.0	35.0 - 45.0 %
MCV	90.5	80.0 - 100.0 fL
MCH	30.6	27.0 - 33.0 pg
MCHC	33.8	32.0 - 36.0 g/dL
RDW	13.7	11.0 - 15.0 %
PLT	300	140 - 400 thousand/uL
MPV	8.7	7.5 - 12.5 fL

**Figure 2:**
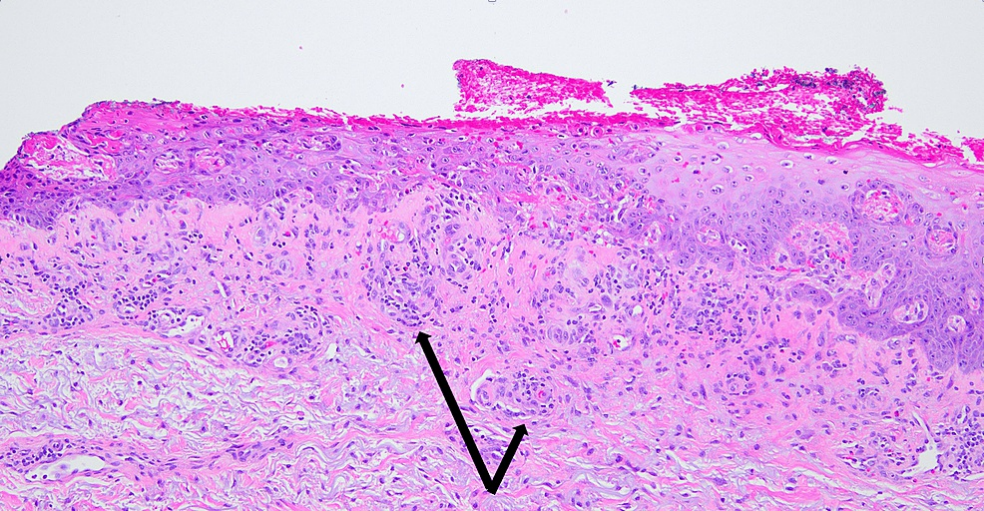
Histopathology of the patient’s punch biopsy showing a superficial perivascular inflammatory infiltrate composed of lymphocytes and eosinophils (examples shown by black arrows) Hematoxylin and eosin (H&E) 5x

**Figure 3:**
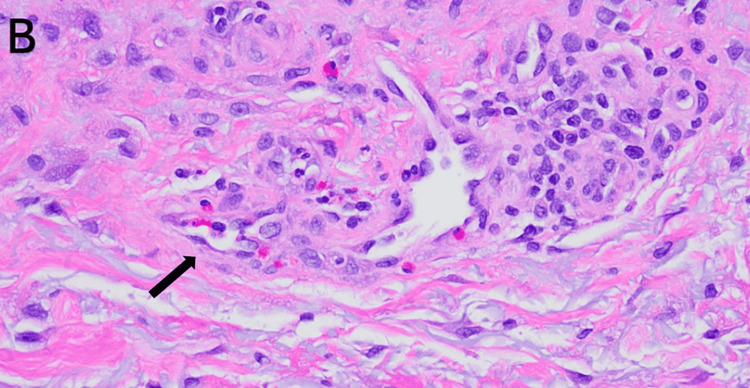
Histopathology of the patient’s punch biopsy showing vacuolar interface dermatitis, with sparsely mixed dermal infiltrate with eosinophils consistent with DRESS Hematoxylin and eosin (H&E) 20x DRESS: drug reaction with eosinophilia and systemic symptoms

The punch biopsy was performed on multiple sites of his right arm. The biopsy specimens were reviewed by a pathologist, showing a vacuolar interface dermatitis, with sparse mixed dermal infiltrate with eosinophils that was consistent with drug reaction with eosinophilia and systemic symptoms (DRESS) syndrome (Figures 2, 3).

Pembrolizumab was discontinued. After treatment with steroids and antivirals for seven days, he improved with decreased rash and blister formation. He was then transitioned from IV acyclovir to oral valacyclovir and cleared for discharge. At the one-month follow-up, the patient presented with an improved appearance of blisters and rashes.

## Discussion

DRESS syndrome is a drug-induced hypersensitivity reaction that is rare, with an incidence of about 1 in 1,000 to 1 in 10,000 after drug exposure [5]. However, DRESS has become more prevalent due to the emergence and advancement of immunotherapy in the treatment of immunologic and neoplastic diseases. Diagnosis can also be challenging due to the broad range of signs and symptoms, leading to lower rates of identification and reports of the condition. The typical presenting symptoms of DRESS include fever, rashes, and eosinophilia; however, they may not all be present. Longstanding eosinophilia has also been associated with systemic damage with neurological, pulmonary, gastrointestinal, and cutaneous changes [5].

In particular, the association between immunotherapy drugs have been greatly associated with DRESS. In addition to the patient in this case, another reported case of pembrolizumab-induced DRESS was described in a patient with hypereosinophilia and neurological changes of mononeuritis with microvascular brain lesions and gastrointestinal changes, such as eosinophilic duodenitis and colitis, approximately one month after the last dose of pembrolizumab [6]. It is important to note that programmed cell death-1 (PD-1) inhibitors, such as pembrolizumab and nivolumab, may cause delayed adverse cutaneous reactions that occur later in treatment compared to cutaneous reactions caused by CTLA-4 inhibitors such as ipilimumab [7]. Another immunotherapy drug linked to DRESS is daclizumab, a monoclonal antibody that is associated with encephalitis and hepatotoxicity [7]. Treatment with sorafenib for hepatocellular carcinoma has also been linked to DRESS with reported side effects of skin reactions and eosinophilia [7].

The main challenges of diagnosing DRESS include the lack of a gold standard for diagnosis and the lack of familiarity with pathology due to its low incidence. The Registry of Severe Cutaneous Adverse Reactions (RegiSCAR) scoring system can be used to make a diagnosis for potential DRESS syndrome cases and requires at least three out of the following four signs: fever greater than or equal to 38.5°C, lymph node enlargement of at least two sites, systemic involvement of at least one internal organ, or blood count abnormalities [7]. However, skin biopsies can also assist with diagnosing cases of DRESS with potential findings of vacuolization, spongiosis, lymphocytic infiltrate, acanthosis, variable presence of eosinophils, or atypical lymphocytes [7]. It is also important to differentiate DRESS from other diseases that involve the skin, such as viral infections as well as other conditions like bullous pemphigoid, and TEN or SJS, as they may have similar presentations. For this patient, it was suspected to be bullous pemphigoid due to the presentation of the lesions as well as several cases in literature involving pembrolizumab-induced bullous pemphigoid. Another theory was that this was a bullous pemphigoid with complications of Herpes zoster as there have been reports of increased risk of developing Herpes zoster in those previously diagnosed with BP [8,9]. There is also a proposed mechanism for DRESS that includes viral reactivation, especially with human herpesvirus (HHV)-6 [9]. However, the punch biopsy for the patient supported a diagnosis of DRESS and was never sent in for direct immunofluorescence as a means for diagnosis of BP. In addition, the primary treatment for DRESS is the discontinuation of the causative drug, depending on severity, and corticosteroids are often used for moderate forms of DRESS [7]. As corticosteroids are also the primary treatment for bullous pemphigoid, it was difficult to discern the true cause of the patient’s presentation before the results of the punch biopsy. However, as the patient’s lesions were progressing, the priority was to treat them based on clinical diagnoses to prevent further complications. As immunotherapy has been a breakthrough in recent years for oncological cases, there has yet to be an effective way to monitor for such adverse skin reactions as the times for the development of the disease and presentations are variable. As the use of these immunotherapy drugs continues to increase, there is still a need to strategize on a standard method of monitoring or even on the means of prevention of such rare skin reactions for those on immunotherapy.

## Conclusions

DRESS is an adverse drug reaction that can be a diagnostic challenge for patients on immunotherapy due to its variety of manifestations and resemblance to other immune-mediated diseases. Prompt recognition of these side effects of immunotherapy is necessary to ensure patient safety. As the current diagnostic tools to immediately evaluate and monitor for such hypersensitivity reactions are insufficient, there is a need for the development of more effective strategies to address these challenges.
